# Professor Dr. G. Jaeger’s Work in America

**Published:** 1893-01

**Authors:** 


### PROFESSOR DR. G. JAEGER'S WORK IN AMERICA.

[Translated from *Allg. Hom. Zeitung*, Vol. 125, p. 159, by B. Fincke, M. D.]

THE HOMŒOPATHIC PHYSICIAN has been publishing for some time the translation of the article of our faithful contributor and investigator, Professor Dr. G. Jaeger, which appears in our Journal this year, together with the curves and tables. Colleague B. Fincke has taken this task upon himself. The Doctor has also read a report on "Neural Analysis, Professor Dr. G. Jaeger's Latest Provings of Homœopathic Potencies." Though he does not agree with Jaeger in the explanation of the *modus operandi* of the high potencies without hinting even at a better explanation, yet he feels himself induced to close his report with the following words, October number, page 428 :

"However, be that as it may, the efforts of our friend, Dr. Jaeger, are of inestimable value, because he furnishes facts and dates in favor of homœopathic potentiation which cannot be silenced nor controverted, for 'numbers prove.'

"Dr. Jaeger complains of the small encouragement which he had from the homœopathic side ever since he published his immortal investigations ten years ago, and as he undergoes the great labor only in the interest of real science and for the best of Homœopathy which he found himself bound to acknowledge as true, after his diligent search for the truth, every homœopathician ought to express freely his appreciation of his noble efforts and his thanks for what he has done for Homœopathy."

In the discussion following, a motion was made and carried to address a letter to Dr. Jaeger by the Secretary, expressing the congratulations and acknowledgments of the Association to the success of his investigations.

In the same number Dr. Goehrum Jaeger defends himself in a short article entitled "Correction" against the reproach that his explanations of the theory of potentiation were too materialistic. (An answer to this has been sent to Leipsic by Dr. B. Fincke, a translation of which shall be given in our next number.)

